# Identification and validation of miR-509-5p as a prognosticator for favorable survival in osteosarcoma

**DOI:** 10.1097/MD.0000000000029705

**Published:** 2022-08-19

**Authors:** Jiekun Guo, Xiang Fang, Jun Zhou, LingGuo Zeng, Bin Yu

**Affiliations:** a Department of Orthopedic Surgery, Nanfang Hospital, Southern Medical University, Guangzhou, China; b Department of Orthopedic Surgery, Yuebei People’s Hospital, Shantou University, Guangdong, China.

**Keywords:** biomarker, immunology, miR-509-5p, osteosarcoma, prognosis

## Abstract

Osteosarcoma (OS) is the most common primary bone cancer diagnosed in children. This study aims to explore the aberrantly expressed miRNAs that are prognostically related and to provide potential biomarkers for the prognosis prediction of OS. The miRNA profiles of OS and adjacent normal controls were obtained from 2 gene expression omnibus cohorts (i.e., GSE28423 and GSE65071). GSE39058 and Therapeutically Applicable Research to Generate Effective Treatments cohorts, which respectively contained 91 and 85 OS samples with both miRNA expression and clinical characteristics, were employed to perform survival and multivariate Cox regression analyses. Lymphocyte infiltration abundance between distinct subgroups was evaluated with the CIBERSORT algorithm and a previously proposed method. Gene set enrichment analysis was used to infer the dysregulated signaling pathways within each subgroup. Of the 31 differentially expressed miRNAs, miR-509-5p (miR-509) was the most significantly prognostic miRNA in the GSE39058 cohort and its high expression was associated with the better OS prognosis (Log-rank *P* = .008). In the Therapeutically Applicable Research to Generate Effective Treatments validation cohort, the association of high miR-509 expression with favorable survival was also observed (Log-rank *P* = .014). The results remained still significant even adjusted for clinical confounding factors in multivariate Cox regression models. Further immunology analyses demonstrated that elevated infiltration of lymphocytes, decreased infiltration of immune-suppressive cells, and immune response-related pathways were significantly enriched in patients with miR-509 high expression. Our study suggests that miR-509 may serve as a potential biomarker for evaluating OS prognosis and provides clues for tailoring OS immunotherapy strategies.

## 1. Introduction

Osteosarcoma (OS), the most common bone tumor, is featured by the immature osteoid and mostly diagnosed in children and adolescents.^[1–3]^ There are approximately 2 million OS patients all over the world,^[4]^ and it accounts for 3 to 5% of newly diagnosed children patients with cancer.^[5]^ Owing to advances of adjuvant chemotherapy and multiple surgical treatment strategies, the 5-year survival of OS patients has dramatically increased to 60 to 70%.^[6–8]^ However, OS patients with metastasis, especially to the lung organ, have an extremely low survival rate of 19% and a high mortality rate.^[8,9]^ Micrometastasis is evaluated to have occurred in 60% of osteosarcoma patients by the first time of examination.^[10]^ Furthermore, a subset of patients would suffer a local relapse and distant metastases after surgery or distinct chemotherapies, and the median survival time of these patients is <1 year.^[11]^ Therefore, identification of early diagnosed and accurately prognostic biomarkers to prolong survival intervals in patients with OS is urgently needed.

Recently, microRNAs (miRNAs), which are broadly present in eukaryotes,^[12]^ have been demonstrated as the potential survival prediction biomarkers and therapeutic targets for several cancer types. They modulate mRNA expression levels of targeted genes at the posttranscriptional phase by binding to the specific regions and exerting the roles of translational inhibition and degradation.^[13]^ To date, multiple studies have reported the association of abnormal expression of miRNAs with the initiation and prognosis of OS, and dysregulated miRNAs were observed in OS tissues and cell lines.^[14,15]^ Elevated hsa-miR-889-3p expression was demonstrated to modulate the cell cycle signaling pathway and further influence OS tumor size in vivo.^[15]^ Fujiwara et al^[16]^ reported that the high-serum concentration of miR-25-3p was associated with the short overall survival in OS patients. In a research published by Roberto et al,^[17]^ miR-138-5p was identified as an intracellular regulator of invasion owing to its increased expression was linked with the worse event-free survival.

The invention of immune checkpoint inhibitors (ICI) has dramatically prolonged the prognosis of advanced/metastatic patients. However, only a fraction of patients could benefit from the ICI treatments. The 3 FDA-approved ICI response biomarkers, including programmed-death ligand-1 (PD-L1) expression,^[18]^ microsatellite instability (MSI),^[19]^ and tumor mutation burden^[20]^ show remarkable effects in clinical practice. Nevertheless, they are sometimes ineffective on assessing ICI efficacy. Novel and stable indicators are needed to better evaluate ICI responses in multiple cancers, including osteosarcoma.

Here, by integrating the publicly available miRNAs profiles and clinical information of OS patients, we aim to identify the novel prognostic biomarkers of miRNAs and to provide evidence for OS survival prediction and immunotherapy guidance.

## 2. Materials and Methods

### 2.1. Clinical information and miRNA profiles collection

The miRNA profiles of OS and adjacent normal controls were acquired from Gene Expression Omnibus cohorts, including GSE28423 and GSE65071. GSE28423 contains 19 OS and 4 normal controls, while GSE65071 with 20 OS and 15 controls. Clinical characteristics (including follow-up information) and miRNA data of 91 OS samples within GSE39058 were used as the training dataset. Therapeutically Applicable Research to Generate Effective Treatments (TARGET) cohort with 85 samples was treated as the validation cohort. The detailed clinical features were shown in Table 1 (Supplemental Digital Content, http://links.lww.com/MD/G971) for GSE39058 and Table 2 (Supplemental Digital Content, http://links.lww.com/MD/G972) for the TARGET cohort. The gene expression profile of 47 OS samples was available in the GSE39058 cohort and was used to perform mRNA-related analyses. Besides, clinical and miRNA data of 260 sarcoma and 436 melanoma samples were acquired from the TCGA cohort. The ethics committee of Nanfang Hospital of Southern Medical University approved the study.

### 2.2. Assessment of tumor-infiltrated immune cells

CIBERSORT method is to use gene expression data and give an evaluation of the abundances of 22 human hematopoietic cell subtypes by employing 547 feature genes from the leukocyte gene signature matrix, termed LM22.^[21]^ The 22 immune cells include 7 T-cell types, naive and memory B cells, plasma cells, NK cells, and myeloid subsets, which play different functions in the tumor microenvironment. Angelova et al proposed an 812-immune-metagene signature to assess 31 distinct immune cells infiltration and tumor immune landscape,^[22]^ specific feature genes for each immune cell type were illustrated in Table 3 (Supplemental Digital Content, http://links.lww.com/MD/G973). We applied both algorithms to achieve comprehensive immune infiltration results.

### 2.3. Microenvironment-based immune-related signatures

Previously revealed representative immune-related signatures that indicating distinct immune statuses were collected as follows: (1) immune and stromal cells signatures, which separately show the total immune and stromal cell infiltration levels in the microenvironment^[23]^; (2) immune cell subsets, enrichment of T cells, B cells, and NK cells^[24]^; (3) IFNγ signature, a signal locates in the central site of antitumor immune and correlates with immunotherapy response^[25]^; (4) T cell–inflamed signature, that consists of 18 inflammatory genes associated with immune response^[26]^; (5) immune cytolytic activity^[27]^; (6) immune signaling molecules^[24]^; (7) cytokines and chemokines.^[24]^ The detailed feature genes for each immune-related signature were exhibited in Table 4 (Supplemental Digital Content, http://links.lww.com/MD/G974).

### 2.4. The signature of activated-stroma

Moffitt et al proposed a stroma-related signature,^[28]^ which was characterized by 2 distinct features; they are activated-stroma (representative genes: *ZNF469*, *VCAN*, *THBS2*, *SULF1*, *SPARC*, *SFRP2*, *POSTN*, *MMP11*, *LUM*, *ITGA11*, *INHBA*, *GREM1*, *FNDC1*, *FN1*, *FAP*, *CTHRC1*, *COMP*, *COL5A2*, *COL5A1*, *COL3A1*, *COL1A2*, *COL1A1*, *COL11A1*, *COL10A1*, and *CDH11*) and normal-stroma (representative genes: *VIT*, *SYNM*, *SCRG1*, *RSPO3*, *RERGL*, *RBPMS2*, *PTX3*, *PLP1*, *OGN*, *MYH11*, *MEOX2*, *LPHN3*, *LMOD1*, *IGF1*, *ID4*, *GPM6B*, *FABP4*, *DES*, *CDH19*, *ANGPTL7*, *ADAMTS1*, *ACTG2*, and *ABCA8*). By using the nearest template prediction algorithm^[29]^ with distinct feature gene subgroups, the activated stromal subtype could be identified.

### 2.5. GSVA and GSEA

Single sample gene set enrichment analysis (ssGSEA) embedded in GSVA package^[30]^ was used to calculate the enrichment scores of all curated immune signatures for each sample based on the feature genes. Differential analysis of expression profile according to distinct miR-509 expression groups was conducted with R package Limma^[31]^ to obtain adjusted P and fold change values. The t values obtained from differential results were subsequently applied to performed gene set enrichment analysis (GSEA) implemented by fgsea package (https://github.com/ctlab/fgsea). The well-curated signaling pathways in hallmark gene sets from the Molecular Signatures Database (MSigDB) were employed as the background signals. The false discovery rate (FDR) and normalized enrichment score (NES) were calculated based on 1 million permutations.

### 2.6. Statistical analyses

All statistical analyses were conducted with R software (version 4.0.2). In this work, OS patients with miR-509 were stratified into high and low-expression subgroups with the median value. Survival curves were achieved with the Kaplan-Meier method and Log-rank test to compare the differences between distinct subgroups. Multivariate Cox regression models within forestmodel package were performed to adjust confounding factors, such as age, sex, and stage. Association of continuous and categorical variables with distinct miR-509 expression subgroups was calculated with Wilcoxon rank-sum test and Fisher exact test, respectively. Two-sided *P* values less than .05 were considered to be statistically significant.

## 3. Results

### 3.1. Differential analysis of miRNAs and identification of miR-509-5p

**Figure 1.**
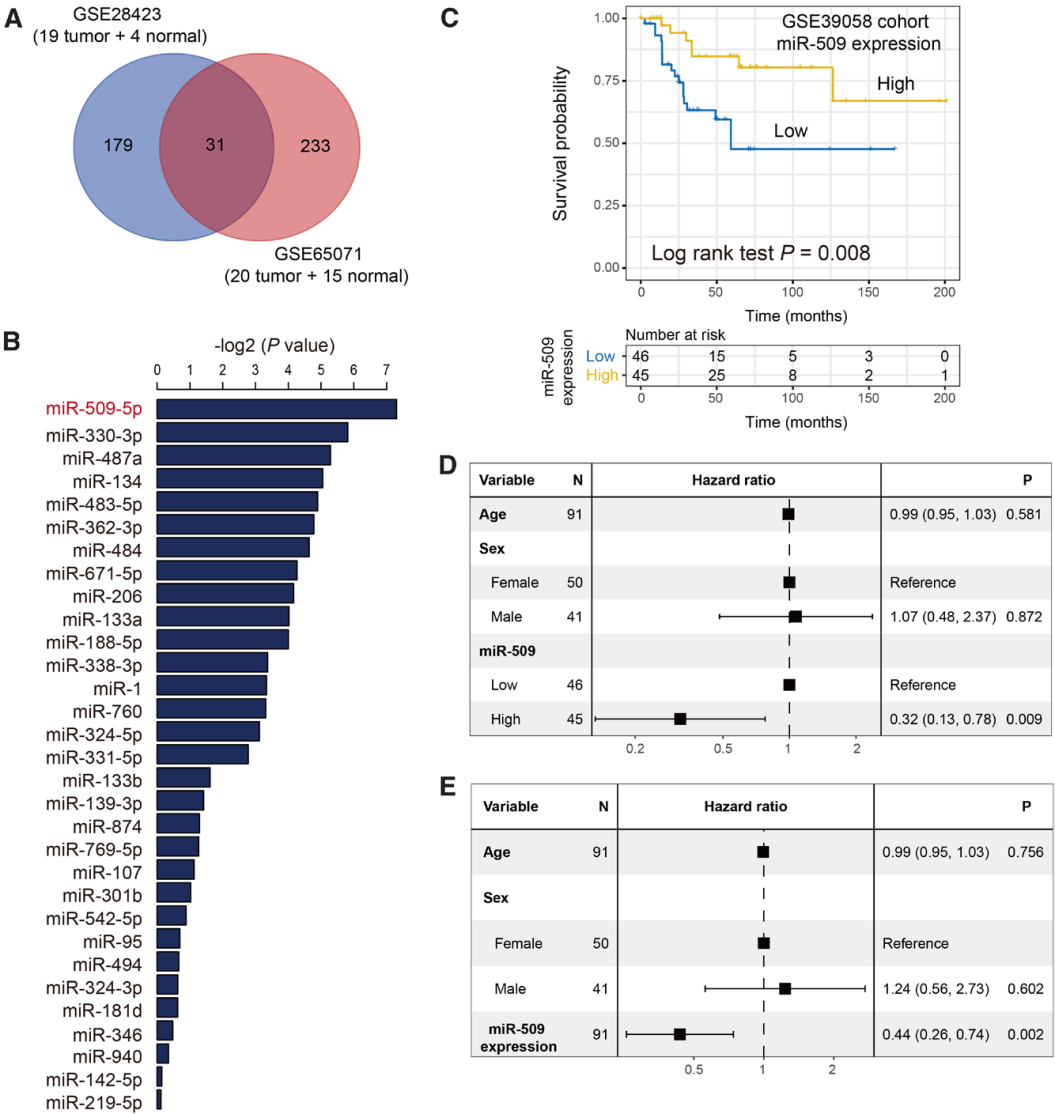
Identification of miR-509-5p as a prognosticator in osteosarcoma. (A) Differential expression analysis based on miRNAs profiles in GSE28423 and GSE65071 cohorts. (B) Prognostic significance of identified 31 miRNAs in GSE39058. (C) Kaplan-Meier survival curves stratified by miR-509 high and low-expression groups. Forest plot representation of Cox regression analysis with clinical confounding factors taken into account in the settings with miR-509 expression regarding as (D) categorical and (E) continuous variables, respectively.

Based on the miRNAs profiles of OS and adjacent normal samples in GSE28423 and GSE65071, we performed the differential analysis of all miRNAs expression. The differential results between tumor and normal samples were illustrated in Table 5 (Supplemental Digital Content, http://links.lww.com/MD/G975) for GSE28423 and Table 6 (Supplemental Digital Content, http://links.lww.com/MD/G976) for GSE65071. We found that a total of 31 miRNAs exhibited the distinct expression in both cohorts (adjusted *P* < .05, |FC| > 2; Fig. 1A). We subsequently performed the Cox regression analysis of the above 31 miRNAs to evaluate their prognostic abilities in GSE39058. The prognosis results were shown in Figure 1B, and we observed that miR-509-5p (miR-509) was the most significant miRNA for assessing OS patients’ survival. OS patients with high miR-509 expression harbored a significantly better survival outcome as compared with those with low expression (Log-rank test *P* = .008; Fig. 1C). This result remained still significant even adjusted for age and sex in the multivariate Cox regression model (HR: 0.32, 95% CI: 0.13–0.78, *P* = .009; Fig. 1D). To obtain a more accurate finding, we treated the miR-509 expression as a continuous variable to conduct multivariate Cox analysis, and the association between miR-509 high expression and favorable prognosis still reached the statistical significance (HR: 0.44, 95% CI: 0.26–0.74, *P* = .002; Fig. 1E).

### 3.2. Validation of the prognostic ability of miR-509 in the TARGET cohort

**Figure 2.**
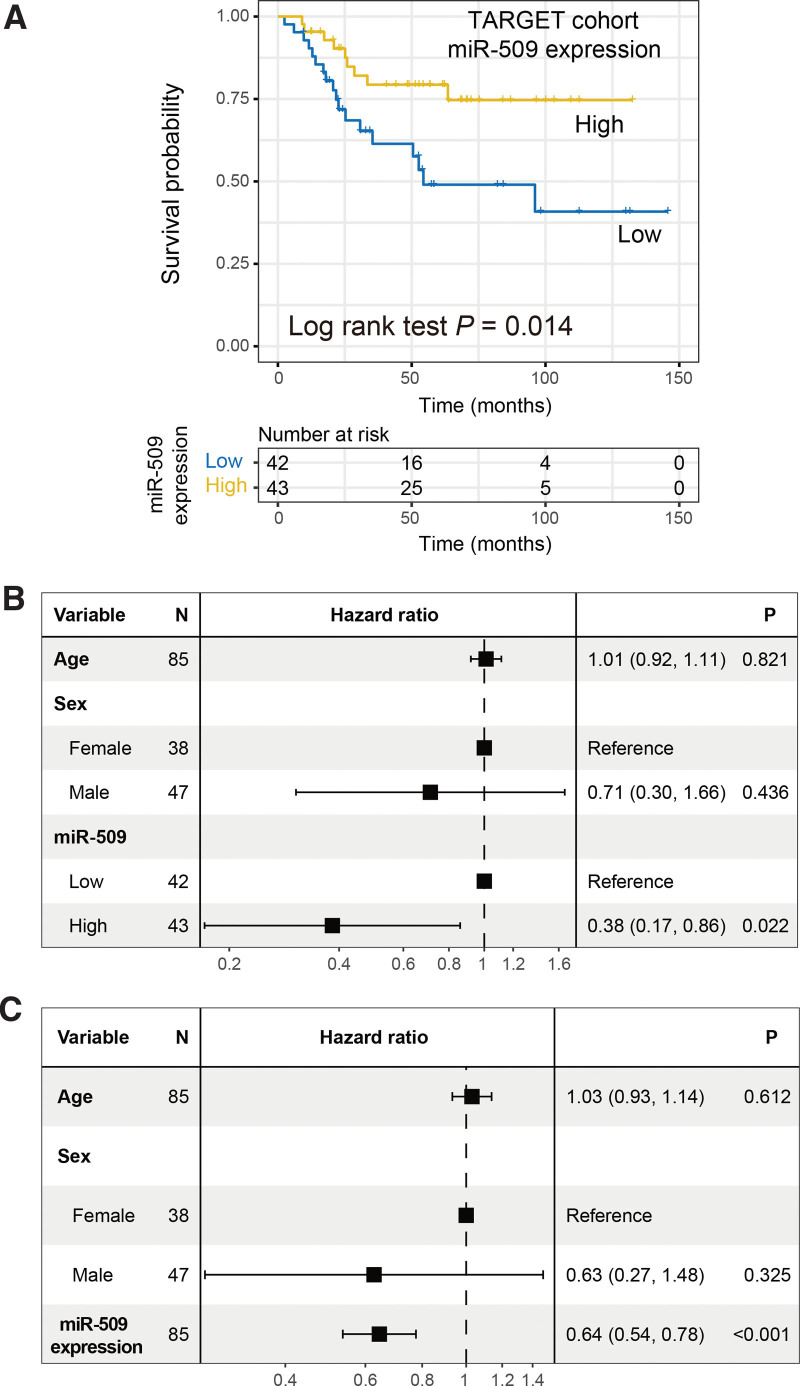
Validation of miR-509 prognostic ability in the TARGET cohort. (A) Kaplan-Meier survival curves stratified by miR-509 distinct expression subgroups. Forest plot representation of Cox regression analysis with age and sex factors taken into account in the settings with miR-509 expression regarding as (B) categorical and (C) continuous variables, respectively.

To obtain a solid link between miR-509 expression and OS prognosis, we employed an independent OS cohort from the TARGET project. In the validation cohort, patients with upregulated miR-509 expression exhibited a markedly preferable survival outcome as compared with the low-expression subgroup (Log-rank test *P* = .014; Fig. 2A). This association remained still significant in the multivariate Cox model with age and sex taken into account (HR: 0.38, 95% CI: 0.17–0.86, *P* = .022; Fig. 2B). We similarly considered the miR-509 expression as a continuous variable and observed the favorable prognosis in patients with the high miR-509 expression (HR: 0.64, 95% CI: 0.54–0.78, *P* < .001; Fig. 2C).

### 3.3. The miR-509 upregulated expression predictive of favorable immune infiltration

**Figure 3.**
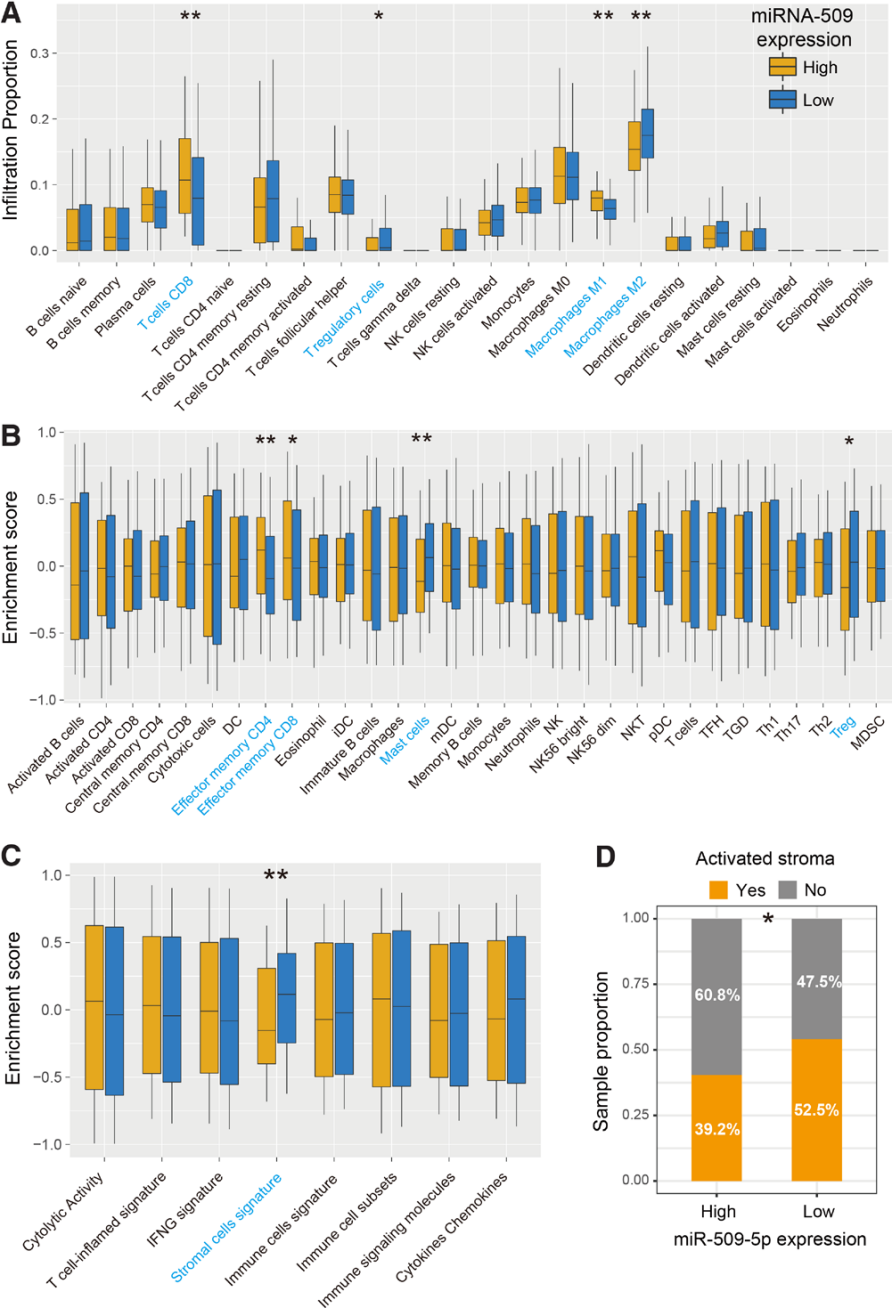
Immunology analyses regarding miR-509 expression. (A) CIBERSORT algorithm and (B) Angelova et al method were employed to infer immune cell infiltration abundance based on distinct miR-509 expression groups. (C) Distinct enrichment scores of collected 8 immune-related signatures in miR-509 upregulated and downregulated subgroups. (D) Distribution of activated-stroma subtype in miR-509 high versus low-expression OS patients.

The aforementioned findings demonstrated the better survival outcome of mIR-509 high expression, we then conducted a series of immunology analyses to explore the potential mechanisms behind the miR-509 regulation. According to the CIBERSORT results, the miR-509 high expression group harbored the significantly increased enrichment of CD8 T cells and M1 macrophages (both *P* < .01; Fig. 3A). Moreover, the immune-suppressive regulatory T cells and M2 macrophages were markedly reductively enriched in the miR-509 upregulated subgroup (both *P* < .05; Fig. 3A). Consistently, with the Angelova et al method, the elevated infiltration of effector memory CD4 and CD8 T cells, and decreased infiltration of regulatory T cells were observed in the miR-509 enhanced expression group (all *P* < .05; Fig. 3B). Noticeably, the mast cells, which were previously revealed as the immune inhibitor,^[32,33]^ were also exhibited reduced infiltration in patients with miR-509 high expression (*P* < .01; Fig. 3B).

Of the 8 collected immune-related signatures, we also performed the differential enrichment analysis between distinct miR-509 expression levels. We observed that the stromal cell signature enrichment, which plays roles in tumor immune escape, was negatively associated with the miR-509 expression (Wilcoxon rank-sum test *P* < .01; Fig. 3C). Further, we calculated the activated-stroma proportion in distinct subgroups based on the Moffitt et al method, and we found that patients with upregulated miR-509 expression had a significantly reduced proportion of activated-stroma subtype (39.2% vs 52.5%, Fisher exact test *P* < .05; Fig. 3D).

### 3.4. Immune response relevant pathways associated with miR-509 expression

**Figure 4.**
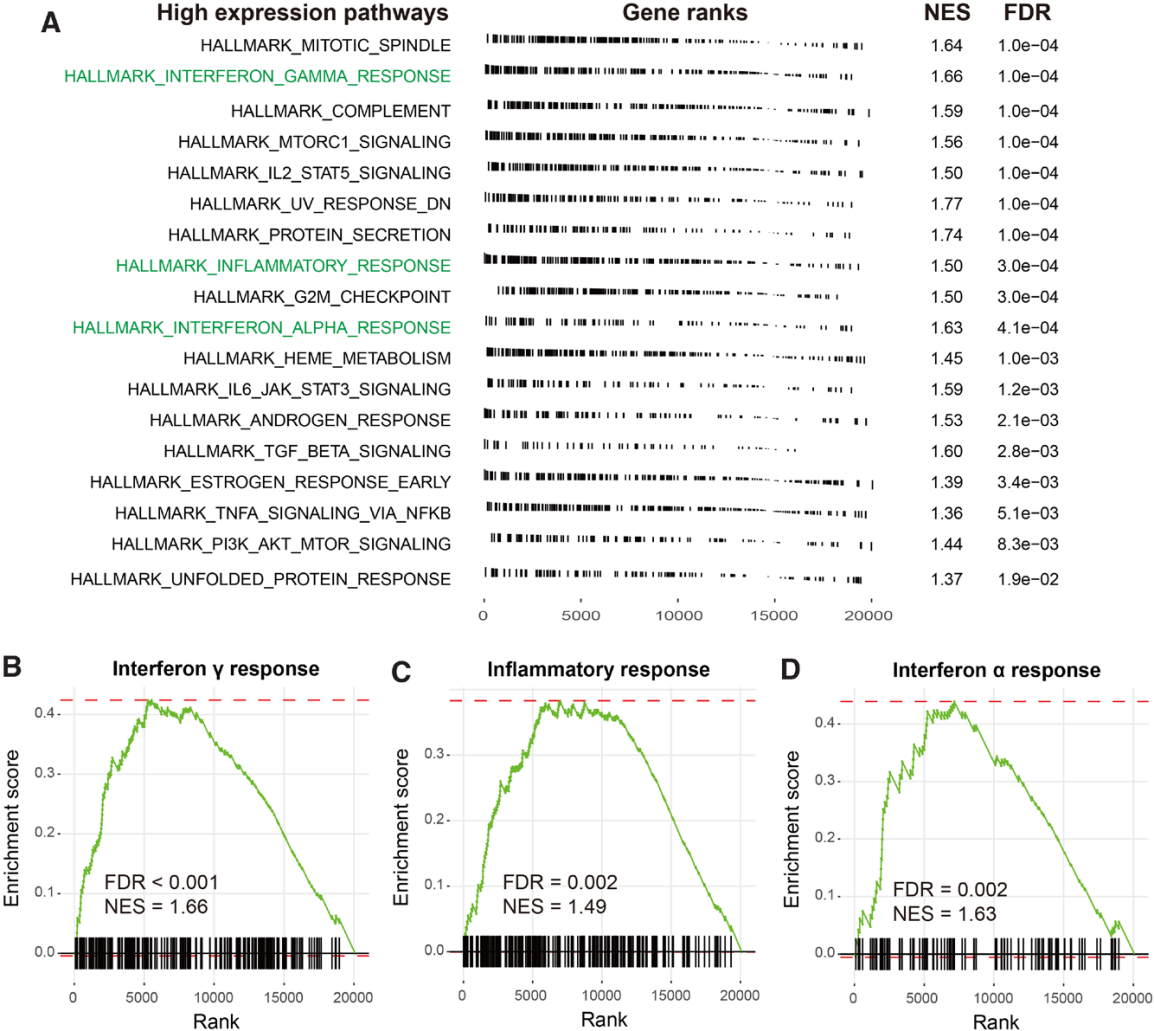
Immune response-related pathways associated with miR-509 expression. (A) GSEA results exhibition for the pathways significantly enriched in the miR-509 upregulated subgroup. Immune response relevant pathways, such as (B) interferon γ response, (C) inflammatory response, and (D) interferon α response were illustrated with GSEA plots. GSEA = gene set enrichment analysis.

By using the gene expression profile from the GSE39058 cohort, we conducted differential expression analysis between 2 miR-509 expression subgroups and extracted t values from the results to performed gene set enrichment analysis (GSEA) against Hallmark gene sets. Results showed that immune response-related signaling pathways, such as interferon γ response (NES = 1.66, FDR < 0.001), inflammatory response (NES = 1.49, FDR = 0.002), and interferon α response (NES = 1.63, FDR = 0.002) were significantly enriched in patients with miR-509 high expression (Fig. 4A). The detailed GSEA enrichment plots were shown in Figure 4B–D.

### 3.5. Further corroboration in TCGA sarcoma and melanoma cohorts

**Figure 5.**
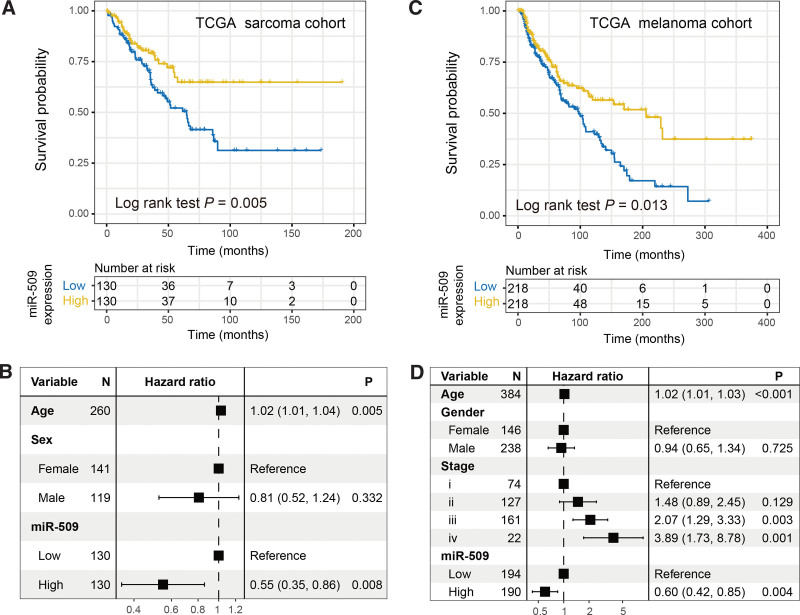
Further corroboration with 2 TCGA cohorts. (A) Kaplan-Meier survival analysis and (B) multivariate Cox regression model were conducted based on distinct miR-509 expression levels in the TCGA sarcoma cohort. (C) Kaplan-Meier survival analysis and (D) multivariate Cox regression model were conducted based on distinct miR-509 expression levels in TCGA melanoma cohort. TCGA = the Cancer Genome Atlas.

To further explore the prognostic implications of miR-509 expression in similar tumors as osteosarcoma, we performed survival and Cox regression analyses regarding miR-509 in TCGA sarcoma and melanoma cohorts. In the TCGA sarcoma cohort, we observed that patients with miR-509 high expression exhibited a significantly improved survival outcome as compared with those low-expression patients in Kaplan-Meier survival analysis (Log-rank test *P* = .005; Fig. 5A) and multivariate Cox regression model (HR: 0.55, 95% CI: 0.35–0.86, *P* = .008; Fig. 5B). Consistently, miR-509 upregulation was also connected with the favorable prognosis in the TCGA melanoma cohort (Log-rank test *P* = .005; Fig. 5C). This association remained still significant after adjusting for age, sex, and stage in the multivariate Cox regression model (HR: 0.60, 95% CI: 0.42–0.85, *P* = .004; Fig. 5D).

## 4. Discussion

We performed a comprehensive analysis of miRNAs and clinical characteristics for osteosarcoma and identified that miR-509-5p high expression was associated with a favorable OS survival outcome. The further immunology analyses revealed that increased infiltration of lymphocytes, decreased infiltration of immune-suppressive cells, and immune response-related signatures and pathways were significantly enriched in the OS patients with miR-509 high expression. Findings derived from this work may provide evidence for OS prognosis prediction and clinical immunotherapy strategies.

The hsa-miR-509-5p could participate in the cancer biological regulation via various signaling pathways. A polymorphism (i.e., rs73092672) in the 3′UTR of miR-509-5p was demonstrated to be associated with a higher risk of breast cancer incidence.^[34]^ The circRNA051239 functioned as a competitive endogenous RNA by sponging miR-509-5p to promote PRSS3 expression, which facilitates proliferation and migration of epithelial ovarian cancer.^[35]^ Sun et al suggested that miR-509-5p may participate in the pathogenesis of male infertility and testicular germ cell tumor through regulating proliferation and apoptosis pathways.^[36]^ Previous studies implicated that miR-509-5p inhibited cellular proliferation and migration by targeting specific genes in pancreatic cancer, ovarian cancer, nonsmall cell lung cancer, and renal cell carcinoma,^[37–40]^ which were consistent with our results that miR-509-5p acted as a tumor suppressor in osteosarcoma.

The miRNAs play their biological functions commonly through targeting a specific gene. The miR-509-5p inhibited cell proliferation, invasion, and migration via targeting the gene of MDM2 in testicular germ cell tumor,^[36]^ pancreatic cancer,^[37]^ cervical cancer,^[41]^ and hepatocellular carcinoma.^[41]^ A recent study reported that miR-509-5p acted as an antioncogene in breast cancer via targeting SOD2.^[42]^ YWHAG and FOXM1 were identified as the target genes for miR-509-5p in nonsmall cell lung cancer.^[39,43]^ The above studies provide the potential targets for miR-509-5p in OS and further careful investigation is necessary.

The classical roles of miRNAs are the regulation of posttranslational biological processes by targeting the 3′UTR or 5′UTR regions of specific genes. The emerging evidence has shown that miRNAs may be also involved in the immune response modulation of cancer. A recent study reported that miR-148a-3p silences the CANX/MHC-I pathway and impairs CD8 T cell-mediated immune attack in colorectal cancer, which provides a rationale for immunotherapy.^[44]^ The miR-30a-5p was identified to regulate the ubiquitination of PD-L1 and inhibit CD8 T-cell response by targeting the USP22 gene, thereby promoting colorectal cancer development.^[45]^ Wang et al demonstrated that miR-329-3p inhibits tumor immunosuppression and reinforces the tumor response to T-cell cytotoxic effect by downregulating KDM1A expression, which contributed to the activation of PD-L1 expression.^[46]^ Based on a lncRNA-miRNA-mRNA regulatory axis, miR-214-3p was revealed to be associated with the decreased B cells, CD8 + T cells, and a worse prognosis of hepatocellular carcinoma.^[47]^ A 9-miRNA signature was also developed to evaluate immune microenvironment and agent responses in gastric cancer.^[48]^ Consistently, in our study, miR-509 upregulated expression predictive of the favorable immune infiltration and causally contributed to the preferable survival outcome in OS, which indicates that miR-509 may be linked with the OS immune regulation.

High expression of miR-509 was identified to link with enhanced infiltration of immune response cells, decreased infiltration of immune-suppressive cells, and immune response signaling pathways in OS. The above findings demonstrated that a hot immune microenvironment (which is an essential condition for a favorable immunotherapy efficacy) was enriched in OS patients with miR-509 high expression. Therefore, we speculate that OS patients with miR-509 high expression may be the potential objectives for receiving immune treatments.

In summary, in this study, miR-509 expression was identified to be associated with the immune infiltration and survival outcome in osteosarcoma. Further mechanistic studies into the functions of miR-509 are needed.

## Author contributions

BY designed this study; JG, XF, JZ, and LZ developed the methodology and acquired the related data; JG, XF, JZ, LZ, and BY performed data analysis and interpretation; JG, XF, JZ, and LZ drafted and revised the article; BY supervised this study. All authors read and approved the final article.

## Supplementary Material


